# Magnesium Lithospermate B Downregulates the Levels of Blood Pressure, Inflammation, and Oxidative Stress in Pregnant Rats with Hypertension

**DOI:** 10.1155/2020/6250425

**Published:** 2020-09-17

**Authors:** Kaixiang Xu, Xiaohong Zang, Mian Peng, Qian Zhao, Binbin Lin

**Affiliations:** ^1^Department of Quality Management, Yangtze River Pharmaceutical (Group) Co., Ltd., Taizhou, Jiangsu 225321, China; ^2^Department of Pharmacy, Taizhou Hospital of Traditional Chinese Medicine, Taizhou, Jiangsu 225300, China; ^3^Chinese Medicine Research Institute, Yangtze River Pharmaceutical (Group) Co., Ltd., Taizhou, Jiangsu 225321, China

## Abstract

**Background:**

Magnesium lithospermate B (MLB) was shown to suppress oxidative stress and reduce hypertension, but the role of MLB in pregnancy-induced hypertension (PIH) remains unknown. The objective of this study was to demonstrate the effects of MLB on rats with PIH.

**Methods:**

A total of 40 pregnant SD rats were selected, and 30 rats were orally given NG-nitro-L-arginine methyl ester (L-NAME, 60 mg/kg/day) to establish PIH rat models. Rats were equally divided into four groups: control, PIH, 5 mg/kg MLB, and 10 mg/kg MLB. MLB was consecutively administered into PIH rats for one week. The effects of MLB on mean arterial blood pressure (MAP), urine protein level, inflammation, and oxidative stress together with angiogenesis were analyzed.

**Results:**

MLB prevented the elevation in MAP and urine protein levels induced by L-NAME. The activities of inflammatory cytokines were highly increased in serum and placental tissues of PIH rats, while cotreatment with MLB partially reversed the activities of these cytokines. MLB also recovered the expression of reactive oxygen species (ROS) in plasma of PIH rats together with levels of oxidative stress and antioxidant capacity in the placenta of PIH rats. The decreased expressions of vascular endothelial growth factor (VEGF), endothelial nitric oxide synthase (eNOS), and NO observed in PIH rats were increased by MLB. In addition, 10 mg/kg MLB exhibited higher protective effects as compared to lower doses of 5 mg/kg.

**Conclusion:**

This study demonstrated that pretreatment with MLB decreased MAP, inflammation, and oxidative stress in rats with gestational hypertension.

## 1. Introduction

Pregnancy-induced hypertension (PIH) is an idiopathic and common disease in obstetrics, and it usually occurs from 20 weeks of gestation to 2 weeks after delivery [1]. The main clinical manifestations of PIH are hypertension, edema, proteinuria, and other signs of preeclampsia (PE), such as hemolytic anemia, elevated liver enzymes, and low platelet count [2], which can be accompanied by functional impairment or dysfunction of multiple organs in the body, including the liver, kidney, brain, and peripheral blood vessels [3]. Women who have a history of PIH are found to have a higher tendency for long-term endothelial dysfunction, higher systolic blood pressure, and low-density lipoprotein cholesterol, compared with women who have not had any pregnancy-related complications [4]. In severe cases of PIH, convulsions, coma, and even death may occur, causing it to be one of the diseases that seriously endanger the safety of mothers and infants [5]. Therefore, it is particularly important to get an early diagnosis for PIH, in order to intervene and carry out a close antenatal surveillance for the treatment of PIH.

However, the cause of PIH has not yet been clarified. Until now, PIH is believed to be related to genetic factors, including immune maladjustment, oxidative stress, capillary endothelial injury, and placental malnutrition [6]. Studies have found that during pregnancy, women will experience some inflammatory response processes, which are physiological stress responses of the body that do not affect or trigger abnormal symptoms of pregnant women. However, the inflammatory factor levels in the placenta or serum of pregnant patients with eclampsia are higher than those of normal pregnant women [7]. Besides, poor functions of the placenta in the early stage will give rise to hypoxia, subsequent inflammatory response, and vascular endothelial system dysfunction, ultimately leading to various clinical manifestations of PE. As important regulators and proinflammatory cytokines during inflammatory responses, interleukin (IL), TNF-*α,* and monocyte chemotactic protein (MCP-1) were found to be significantly overexpressed in the plasma of PIH women compared with those in the plasma of normal pregnant women [8].

Recent research has reported that oxidative stress plays a pivotal role in the progression of PE, and elevated levels of antiangiogenic factors are associated with PIH [7, 8]. Alterations in the proportion of proangiogenic factors, including vascular endothelial growth factor (VEGF) and antiangiogenic cytokines, can lead to angiogenic imbalance. Subsequently, inflammatory responses and oxidative stress can be triggered by the disrupted angiogenic balance and altered regulation of various factors, eventually resulting in endothelial dysfunction and hypertension [9]. As a result, identifying the molecular mechanism underlying and developing therapeutic strategies that can alleviate maternal inflammation, oxidative stress, and endothelial dysfunction are extremely important for treating PIH.

Magnesium lithospermate B (MLB), an active component of the water-soluble fraction of *Salvia miltiorrhiza*, is noted for scavenging oxygen free radicals, protecting cell mitochondria, improving energy metabolism, and regulating gene expression. The protective effects of MLB on cerebral/hepatic/myocardial ischemia/reperfusion injury and renal injury together with vascular endothelial dysfunction via regulating inflammation and oxidative stress have been extensively demonstrated [10–14]. In another report, MLB was shown to prevent phenotypic transformation of pulmonary arteries with pulmonary hypertension and alleviate sodium-induced hypertension and renal failure in rats [15, 16]. Based on these findings, we predict that MLB can prevent the development of PIH by inhibiting inflammation and oxidative stress.

In the present study, by using an NG-nitro-L-arginine methyl ester- (L-NAME-) induced PIH rat model, we investigated whether MLB was able to prevent the increase of MAP and proteinuria and whether the preventive effect of MLB is related to suppression of inflammation and oxidative stress.

## 2. Materials and Methods

### 2.1. Ethical Statement

The experiments of animals have declared to follow the principles of the World Medical Association (WMA) Declaration of Helsinki.

### 2.2. Reagents and Animals

Magnesium lithospermate B (MLB) was purchased from Shanghai Green Valley Pharmaceutical Company and dissolved in normal saline. NG-nitro-L-arginine methyl ester (L-NAME) was purchased from Abcam Biotechnology and dissolved in normal saline.

30 male (weight, 280–320 g) and 60 female (weight, 220–270 g) specific pathogen-free (SPF) Sprague Dawley (SD) rats were purchased from Nanjing Junke Biological Engineering Co., Ltd., and kept under a temperature- and humidity-controlled condition with a 12 h light/12 h dark cycle in the present study.

### 2.3. Establishment of Pregnancy-Induced Hypertension Rat Model

After keeping rats for one week, male and female (1 : 2) rats were kept in one cage to allow them to mate. On the second day, the vaginal secretions of female rats were collected and examined under a microscope, and the presence of sperm was considered as 0^th^ day of pregnancy.

Pregnant rats were normally bred until day 15 of pregnancy, and then a total of 40 pregnant female rats were equally assigned to four groups (10 rats each group) and additionally received indicated reagents besides normal diet: (і) control, rats were fed with normal saline; (ii) PIH, rats were orally given 60 mg/kg/day L-NAME for one week [17]; (iii) MLB 5 mg/kg, rats were orally given 60 mg/kg/day L-NAME and intraperitoneally administrated 5 mg/kg/day MLB for one week; and (iv) 10 mg/kg MLB, rats were orally given 60 mg/kg/day L-NAME and intraperitoneally administrated 10 mg/kg/day MLB for one week.

On day 21 of pregnancy, the aortal blood of rats was collected, the rats were anaesthetised, and hysterotomies were performed to collect placental tissues. After the surgery, all rats were sacrificed by intraperitoneal administration with 100 mg/kg phenobarbital.

### 2.4. Measurement of Blood Pressure and Urine Proteins

The mean arterial blood pressure (MAP) was recorded on days 15–21 of pregnancy using the CODA 6 BP system (Kent Scientific, Torrington, CT, USA).

The content of 24 h urine proteins was quantified by a urine protein test kit (Nanjing Jiancheng Biological Engineering Institute) after collecting 24 h urine. Briefly, from day 15 of gestation, pregnant rats were kept in an automated home cage system (PhenoMaster, Beijing, China). For measuring MAP, rats were catheterized under isoflurane anesthesia (Webster, Sterling MA). The carotid artery was cannulated, and MAP was recorded continuously for a 2 h period after 1 h of stabilization. The urine can be automatically accumulated in the system and was collected every 24 h.

### 2.5. Enzyme-Linked Immunosorbent Assay (ELISA)

The whole blood collected from the aorta of rats was placed at room temperature for 2 h and then centrifuged at 2500 rpm for 10 min. The placental tissues were homogenized in PBS on ice. The placental homogenates were frozen in liquid nitrogen and stored at −80°C until further processing. The upper serum was collected and stored at −80°C for use. ELISA kits (Abcam Biotechnology) were utilized to detect the activity of TNF-*α* (cat. no. ab100785), IL-1*β* (cat. no. ab100768), IL-6 (cat. no. ab100772), and monocyte chemotactic protein (MCP-1) (cat. no. ab219045) in plasma according to the manufacturer's protocol.

### 2.6. Assessment of Antioxidant Capacity and Oxidative Stress

The level of reactive oxygen species (ROS) in rats' plasma was analyzed by a cellular ROS assay kit (Abcam Biotechnology, cat. no. ab113851) according to the manufacturer's protocol.

The placental tissues were washed with PBS, dissolved with a cell lysing solution containing protease inhibitor, and then subjected to ultrasonic treatment to fully lyse the samples. After centrifugation, supernatants were collected, and the protein concentrations were determined using the Bio-Rad bicinchoninic acid (BCA) assay kit (Bio-Rad, Hercules, CA, USA).

The antioxidant capacity of the placental tissue was measured using the Trolox equivalent antioxidant capacity (TEAC) assay kit (Cayman Chemical Company, Ann Arbor, MI, USA), and the levels of thiobarbituric acid reactive substances (TBARS) and NO were determined using the TBARS and nitrate/nitrite colorimetric assay kit (Cayman Chemical Company) according to the manufacturer's instructions, respectively.

### 2.7. RNA Isolation and Reverse Transcription-Quantitative PCR (RT-qPCR)

The total RNA of placental tissues was extracted using TRIzol® reagent (Invitrogen, Thermo Fisher Scientific, Inc.), and 1 *μ*g total RNA was converted to cDNA using the PrimeScript™ RT reagent kit with gDNA Eraser (Takara Biotechnology Co., Ltd.) according to the manufacturer's protocols. The following RT temperature protocol was used: 37°C for 15 min and 85°C for 5 sec. qPCR was performed using TB Green® Fast qPCR Mix (Takara Biotechnology Co., Ltd.) according to the manufacturer's protocols. Differential expression of mRNA was calculated using the 2^−ΔΔCq^ method (24). The primers used were as follows: VEGF, forward 5′-ACCATGAACTTTCTGCTC-3′and reverse 5′-GGACGGCTTGAAGATATA-3′; GAPDH, forward 5′-AGTGCCAGCCT-CGTCTCATA-3′ and reverse 5′-TGAACTTGCCGTGGGTAGAG-3′. The following thermocycling conditions were used for qPCR: initial denaturation at 95°C for 30 sec and 40 cycles of 95°C for 5 sec and 60°C for 15 sec, followed by default of melt curve (Applied Biosystems 7500; Thermo Fisher Scientific, Inc.).

### 2.8. Western Blot Analysis

After quantifying, equal amounts (50 *μ*g) of proteins were separated by 8–12% SDS-PAGE. Separated proteins were transferred onto PVDF membranes (Bio-Rad Laboratories, Inc.) and then blocked with 5% nonfat milk at 37°C for 1 h, after which the membranes were incubated with the following primary antibodies (Abcam) overnight at 4°C: VEGF (cat. no. ab32152), eNOS (cat. no. ab76198), and anti-GAPDH (cat. no. ab32152). Finally, the membranes were incubated with a horseradish-conjugated secondary antibody (goat anti-rabbit IgG; cat. no. ab205718; 1 : 10,000) at room temperature for 2 h and visualized by an electrochemiluminescence system (Amersham Imager 600; GE Healthcare). Image J software (1.8.0_112, National Institutes of Health) was utilized for densitometric analysis of western blot.

### 2.9. Statistical Analysis

All data are expressed as the mean ± standard deviation, and the statistical analyses were conducted using GraphPad Prism 6 (GraphPad Software, Inc.). Differences were calculated by Student's *t*-test or one-way ANOVA followed by Turkey's post hoc test. *P* < 0.05 was considered to indicate statistically significant differences.

## 3. Results

### 3.1. MLB Decreases the Elevated Level of Arterial Blood Pressure and Urine Proteins in PIH Rats

**Figure 1:**
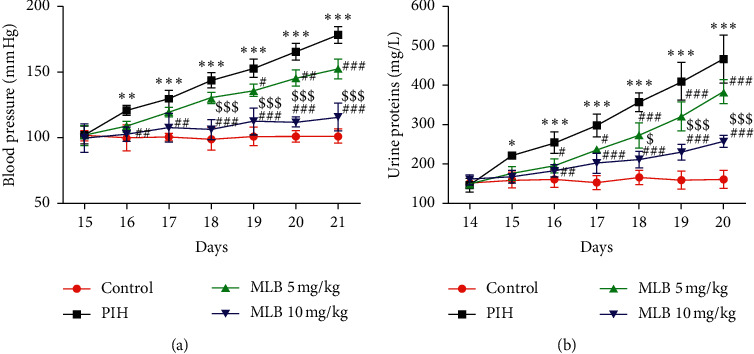
MLB decreased MAP and urine proteins. (a) MAP recorded from day 15 to day 21 of pregnancy (*n* = 10). (b) The contents of 24 h urine proteins measured from day 15 to day 21 of pregnancy (*n* = 10). ^*∗*^*P* < 0.05, ^*∗∗*^*P* < 0.01, and ^*∗∗∗*^*P* < 0.001 vs. control. ^#^*P* < 0.05, ^##^*P* < 0.01, and ^###^*P* < 0.001 vs. PIH. ^$$$^*P* < 0.05 and ^$$$^*P* < 0.001 vs. MLB 5 mg/kg. MAP, mean arterial blood pressure; MLB, magnesium lithospermate B; PIH, pregnancy-induced hypertension.

First of all, to observe the effects of MLB on MAP and urine proteins in PIH rats, we measured MAP and collected 24 h urine to detect the content of urine proteins from 15–21 days of pregnancy. Results showed that normal control pregnant rats maintain a stable blood pressure and urine proteins, while PIH-treated rats had elevated MAP and urine proteins in a time-dependent manner from day 16 (the second day of L-NAME treatment) to day 21 of pregnancy (Figure 1). The mean MAP and level of urine proteins recorded for MLB-treated PIH rats were lower than those recorded for the PIH group from day 17 of pregnancy until the last day of the experiment (day 21 of pregnancy). Besides, as revealed in Figure 1, a higher dose of MLB (10 mg/kg) decreased MAP and urine proteins more effectively than the lower dose did (5 mg/kg).

### 3.2. MLB Prevents the Generation of Inflammatory Cytokines in Both the Serum and Placenta Tissue of PIH Rats

**Figure 2:**
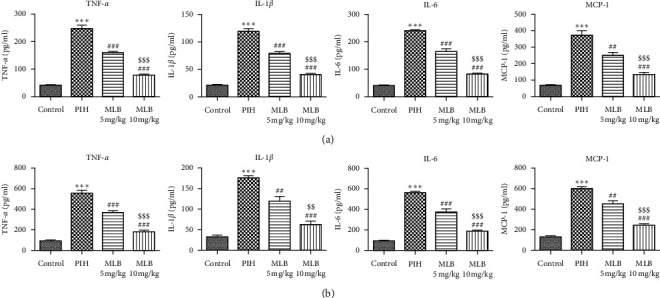
MLB inhibited generation of inflammatory cytokines. (a) The levels of TNF-*α*, IL-1*β*, IL-6, and MCP-1 in serum of rats (*n* = 10). (b) The levels of TNF-*α*, IL-1*β*, IL-6, and MCP-1 in placentas of rats (*n* = 10). ^*∗∗∗*^*P* < 0.001 vs. control. ^##^*P* < 0.01 and ^###^*P* < 0.001 vs. PIH. ^$$^*P* < 0.01 and ^$$$^*P* < 0.001 vs. MLB 5 mg/kg. MLB, magnesium lithospermate B; PIH, pregnancy-induced hypertension; TNF-*α*, tumor necrosis factor-*α*; IL, interleukin; MCP, monocyte chemotactic protein.

Then, we extracted the serum and placenta tissue of rats to detect the activity of inflammation. As shown in Figure 2, L-NAME treatment (PIH group) caused a significant increase in concentrations of TNF-*α*, IL-1*β*, IL-6, and MCP-1 in both the serum and placenta of pregnant rats compared with that of normal pregnant rats, indicating the enhancement of inflammatory response. However, the cotreatment of MLB partially reduced the increased activity of these inflammatory cytokines in PIH rats. Similarly, MLB with the dose of 10 mg/kg possessed better inhibitory ability against inflammation.

### 3.3. MLB Reduces Oxidative Stress in PIH Rats

**Figure 3:**
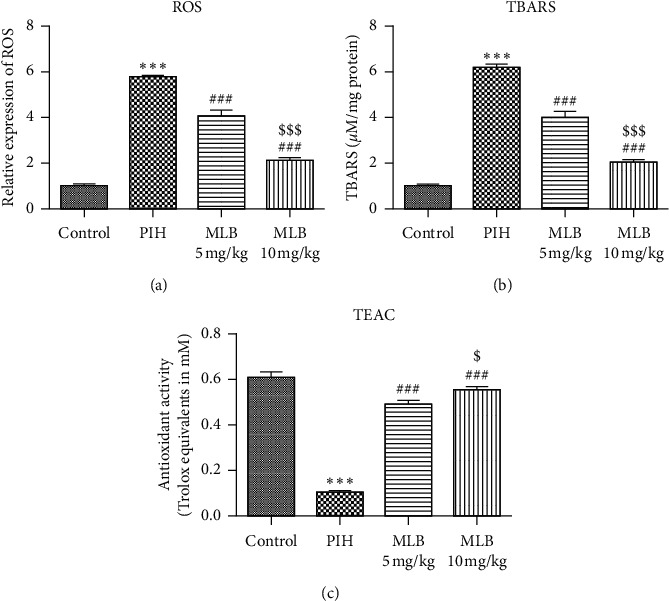
MLB suppressed oxidative stress: (a) relative expression of ROS in each group (*n* = 10). (b, c) The levels of TBARS (b) and TEAC (c) in placentas of each group, respectively (*n* = 10). ^*∗∗∗*^*P* < 0.001 vs. control. ^###^*P* < 0.001 vs. PIH. ^$^*P* < 0.05 and ^$$$^*P* < 0.001 vs. MLB 5 mg/kg. MLB, magnesium lithospermate B; PIH, pregnancy-induced hypertension; ROS, reactive oxygen species; TBARS, thiobarbituric acid reactive substances; TEAC: Trolox equivalent antioxidant capacity.

Next, the effects of MLB on oxidative stress in PIH rats were explored. The levels of ROS and TBARS, which are both oxidative stress markers, were observed to be highly increased in PIH rats, but their levels visibly dropped upon the presence of MLB (Figures 3(a) and 3(b)). At the same time, MLB administration considerably improved the TEAC level in placental tissues, where the TEAC level was decreased in the PIH group (Figure 3(c)). The level of TEAC mirrors the antioxidant capacity of the placental tissue, these results thus indicating the efficacy of MLB.

### 3.4. MLB Rescues the Angiogenic Ability of PIH Rats

**Figure 4:**
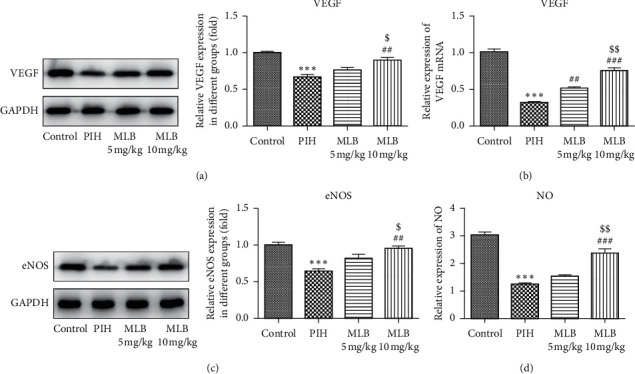
MLB rescued levels of VEGF and NO. (a) Representative immunoblot analysis together with relative protein expression of VEGF in placentas of each group (*n* = 10). (b) Relative mRNA expression of VEGF in placentas of each group (*n* = 10). (c) Representative immunoblot analysis together with relative protein expression of eNOS in placentas of each group (*n* = 10). (d) Relative expression of NO in placentas of each group (*n* = 10). ^*∗∗∗*^*P* < 0.001 vs. control. ^##^*P* < 0.01 and ^###^*P* < 0.001 vs. PIH. ^$^*P* < 0.05 and ^$$^*P* < 0.01 vs. MLB 5 mg/kg. MLB, magnesium lithospermate B; PIH, pregnancy-induced hypertension; VEGF, vascular endothelial growth factor; eNOS, endothelial nitric oxide synthase; NO, nitric oxide.

Finally, we investigated the effects of MLB on angiogenic ability. The imbalance of angiogenesis is strongly associated with the etiology of hypertension and PIH [18]. VEGF and vasodilator NO, which can be synthesized by eNOS, can function as a promoter of angiogenesis. As shown in Figure 4, the expressions of VEGF protein (Figure 4(a)), mRNA (Figure 4(b)), and eNOS protein (Figure 4(c)) together with the activity of NO (Figure 4(d)) were significantly decreased in PIH rats. The supplementation of 5 mg/kg MLB slightly enhanced the level of these cytokines while 10 mg/kg MLB considerably enhanced the level of these cytokines.

## 4. Discussion

PIH comprises of preeclampsia, eclampsia, and high MAP, all of which occurs especially during pregnancy [19]. High MAP and proteinuria, the amount of which indicates the severity of PIH, are the main clinical manifestations of PIH. The increase of urinary protein level, which can reflect the degree of glomerular damage, can also directly damage glomerular mesangial cells, aggravate kidney damage, and cause renal dysfunction [20]. The etiology of PIH is complicated, while endovascular damage and dysfunction caused by excessive oxidative stress, which in turn triggers a series of oxidative stress and inflammatory reactions, are the mechanisms that have been more extensively studied. Therefore, this study aimed to investigate the effects of MLB on MAP, urinary protein content, inflammation, and oxidative stress, as well as angiogenic ability of PIH rats, in order to explore the protective effect of MLB on PIH.

Produced by vascular endothelial cells, NO has been proven to regulate vascular tension, dilate blood vessels, and function as a regulator of the cardiovascular system during pregnancy [21]. L-NAME is an inhibitor against NO synthesis, so the use of L-NAME to establish a PIH model in this study is in line with the theory that vascular endothelial cell damage is the central link in the pathogenesis of PIH, and is also a publicly accepted PIH model [22–24]. In humans, PIH usually occurs during the third trimester, after 20 weeks of pregnancy. To reflect this, L-NAME was first administered on day 15 of rats' pregnancy, considering the gestation period of SD rats is approximately 21 days. On the second day of L-NAME administration, the levels of MAP and urine proteins were significantly increased compared with normal pregnant rats, and L-NAME enhanced MAP and urine proteins in a time-dependent manner from day 15 to day 21 of pregnancy (Figure 1), indicating the availability of our PIH models. At the same time, MLB with the dose of 10 mg/kg considerably reduced the levels of MAP and urine proteins in PIH rats, and significant effects of a lower dose of MLB (5 mg/kg) on MAP and urine proteins displayed from day 19 and day 17 of pregnancy, respectively (Figure 1). These results provided evidence for the protective effect of MLB on PIH.

Considering the mechanism that excessive oxidative stress can cause endovascular damage, thereby triggering a series of oxidative stress and inflammatory reactions, eventually leading to PIH-related symptoms, we next investigated the alteration of cytokines involved in inflammation, oxidative stress, and angiogenesis. In fact, the inhibitory effects of MLB on inflammation and oxidative stress have been extensively demonstrated. The finding by Gao et al. [11] was that pretreatment with MLB inhibited upregulation of inflammatory cytokines intercellular cell adhesion molecule (ICAM-1), vascular cell adhesion molecule (VCAM-1), and TNF-*α*, thereby alleviating LPS-induced endothelial dysfunction in a murine model of acute inflammation. Another report showed the reduced release of TNF-*α*, IL-1*β*, and IL-6 in myocardial ischemia/reperfusion (MI/R) rats treated with MLB [25]. Consistent with others' findings, we confirmed that MLB functions as an inhibitor against inflammation in both the serum and placenta of PIH rats (Figure 2). All the inflammatory markers were significantly increased in PIH, indicating that women who developed PIH later had an inflammatory milieu from the beginning of pregnancy [26]. Inhibition of inflammatory response has been suggested to relieve PIH [27]. Our results suggested that MLB may prevent the occurrence of PIH via inhibiting the inflammatory response. Oxidative stress was shown to enhance in patients with PIH, as well as in animal models of PIH [28]. In the current study, we also illustrated that MLB could blunt L-NAME-induced increase of oxidative stress in the placenta of pregnant rats (Figure 3). NO can be synthesized by eNOS, and it plays a pivotal role in the homeostasis of vasodilation during a normal pregnancy. VEGF is involved in both vasculogenesis and angiogenesis, which are critical for the development of the placenta. Previous study suggested that MLB can restore the microcirculation dysfunction via activating eNOS, and in turn, enhancing the vascular NO production [10]. In the present study, MLB also rescued the expressions of VEGF and NO, indicating the protective effect of MLB on the placental development.

## 5. Conclusion

Taken together, this study demonstrated for the first time that MLB exerted the preventive effect on PIH via reducing inflammation and oxidative stress, regulating the expressions of VEGF and NO, thereby restoring MAP and urine proteins in PIH rats. Therefore, MLB could potentially be used in the clinic in the third trimester for pregnant women, especially pregnant women with a history of hypertension, to prevent PIH. However, further studies will be required to uncover the underlying molecular mechanisms involved.

## Data Availability

All data generated or analyzed during this study are included in this published article.
